# A reconstructed melanoma data set for evaluating differential treatment benefit according to biomarker subgroups

**DOI:** 10.1016/j.dib.2017.05.005

**Published:** 2017-05-05

**Authors:** Jaya M. Satagopan, Alexia Iasonos, Joseph G. Kanik

**Affiliations:** Department of Epidemiology and Biostatistics, Memorial Sloan Kettering Cancer Center, New York, NY, USA

## Abstract

The data presented in this article are related to the research article entitled “Measuring differential treatment benefit across marker specific subgroups: the choice of outcome scale” (Satagopan and Iasonos, 2015) [1]. These data were digitally reconstructed from figures published in Larkin et al. (2015) [2]. This article describes the steps to digitally reconstruct patient-level data on time-to-event outcome and treatment and biomarker groups using published Kaplan-Meier survival curves. The reconstructed data set and the corresponding computer programs are made publicly available to enable further statistical methodology research.

**Specifications Table**

**Table:** 

Subject area	*Biostatistics*
More specific subject area	*Clinical Biostatistics*
Type of data	*Text file*
How data was acquired	*Digital extraction techniques and statistical methods using Adobe Illustrator**[3]**, DigitizeIt software package**[4]**and the R programming language**[5]*
Data format	*Raw*
Experimental factors	*A total of 843 melanoma patients with positive or negative programmed death 1 ligand expression were randomized to receive nivolumab monotherapy, ipilimumab monotherapy or combination therapy. The study has 6 subgroups defined by 3 treatments and two levels of programmed death 1 ligand expression.*
Experimental features	*Individual patient data were extracted from Kaplan-Meier figures and the number at risk reported below the figures for each of the 6 subgroups*
Data source location	*Kaplan-Meier figures published in Figs. 1B and 1C of Larkin et al.**[2]*
Data accessibility	*The reconstructed data and R functions are available at*https://www.mskcc.org/sites/default/files/node/137932/documents/2017-04-20-14-31-36/dataexample.zip
Related research article	*J. M. Satagopan, A. Iasonos, Measuring differential treatment benefit across marker specific subgroups: the choice of outcome scale, Contemp Clin Trials.**[1]*

**Value of the data**

• The data set presents reconstructed information on progression free survival in metastatic melanoma patients and could be used by other researchers.
• This reconstructed data set allows other researchers to develop statistical methodologies for evaluating differential treatment benefit according to biomarker level.
• This reconstructed data set allows other researchers to extend the statistical analyses and compare the results to other similar studies.

## 1 Data

We present reconstructed data based on Fig. 1B and C of Larkin et al. [2]. The reconstructed data set includes information on time to disease progression, progression status, treatment, and the status of programmed death 1 ligand expression for 843 metastatic melanoma patients: 620 with negative expression (210 randomized to the combination therapy arm, 202 to ipilimumab monotherapy and 208 to nivolumab monotherapy) and 223 with positive expression (68 randomized to the combination therapy arm, 75 to ipilimumab monotherapy and 80 to nivolumab monotherapy). The reconstructed data are only approximate data to facilitate statistical methodology research, and do not represent actual patient-level data. These reconstructed data are new and original in the sense that the reconstructed time to progression free survival and progression status data has not been published elsewhere.

## 2 Experimental design, materials and methods

**Fig. 1:**
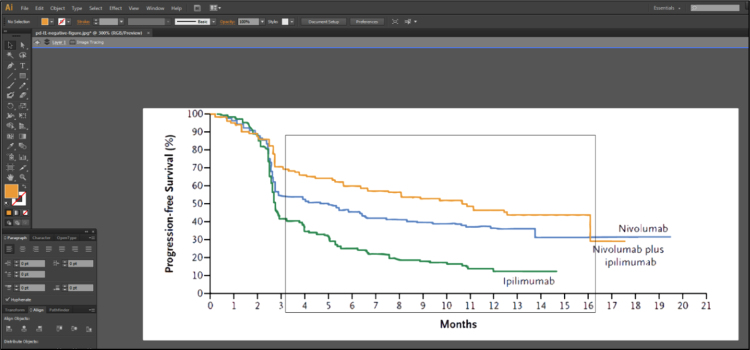
Fig. 1C of Larkin et al. [2] imported into Adobe Illustrator.

We used the following steps to reconstruct data from Figs. 1B and 1C of Larkin et al. [2].

**Step 1: Isolating individual lines from Kaplan-Meier figures**

**Fig. 2:**
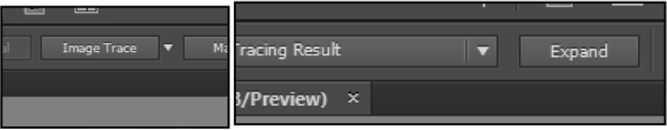
Select the overall image and head to the top option to “Image Trace”, selecting the arrow on the right and choosing “High Fidelity Photo”. Next, select the button on the right of where Image Trace was, “Expand”.

**Fig. 3:**
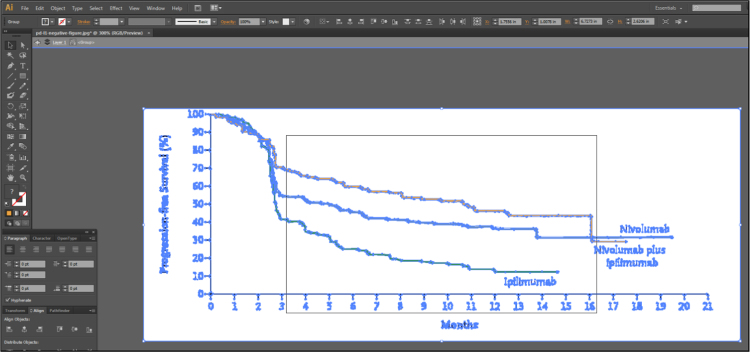
The figure in Adobe Illustrator after expanding via Image Trace.

**Fig. 4:**
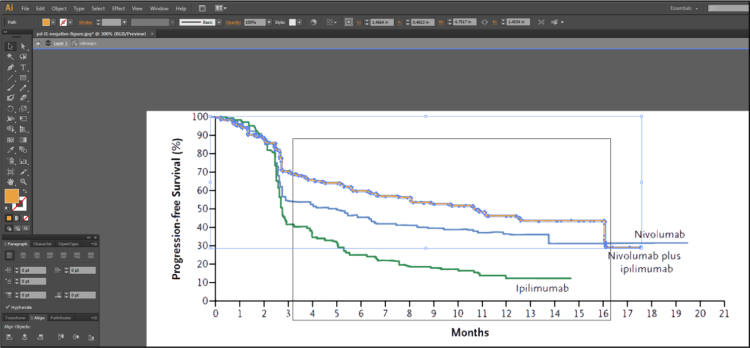
It is now possible to select each line with just a click of the button. Because the trace was for a “High Fidelity Photo”, Adobe Illustrator is able to understand that left clicking an orange line should highlight the entirety of the orange line and nothing else as displayed in this figure. Now, each line can be removed to obtain separate files for each line of data.

**Fig. 5:**
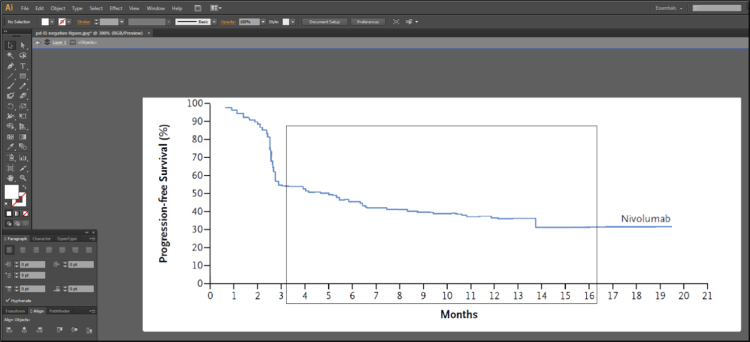
The isolated Nivolumab line in Adobe Illustrator.

**Fig. 6:**
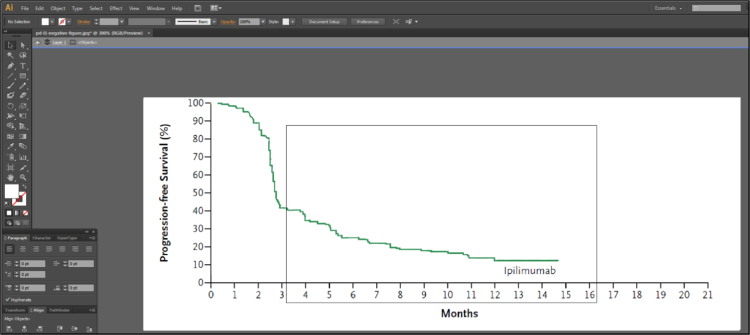
The isolated Ipilimumab line in Adobe Illustrator.

**Fig. 7:**
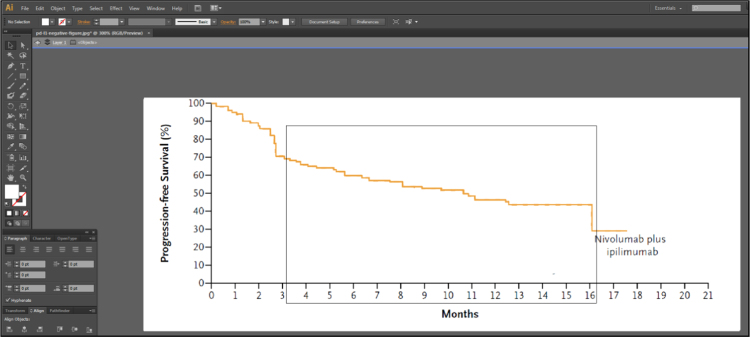
The isolated Nivolumab plus Ipilimumab (combination therapy) line in Adobe Illustrator.

Fig. 1C of Larkin et al. [2] contains 3 lines representing the Kaplan-Meier estimates of survival probabilities for patients with negative programmed death 1 ligand expression randomized to nivolumab monotherapy, ipilimumab monotherapy and combination therapy. Isolate these 3 lines using Adobe Illustrator [3], as described in Fig. 1, Fig. 2, Fig. 3, Fig. 4, Fig. 5, Fig. 6, Fig. 7. Use similar methods to isolate the 3 lines from Fig. 1B of Larkin et al. [2] that correspond to patients with positive programmed death 1 ligand expression. Save the isolated lines as separate jpeg files.

**Step 2: Digital extraction of time and survival probabilities**

Consider a jpeg file containing a single line – for example, the jpeg file corresponding to Fig. 7. Launch the DigitizeIt software package [4] in your computer and open this jpeg file. To digitize the line, select the desired minimum and maximum points on the horizontal (i.e., x) and vertical (i.e., y) axes, click the “Line” icon and left click the mouse on any part of the line. This will digitize the line and show the times (x-axis) and survival probability estimates (y-axis) in the output frame, which can be saved as a text file. The demo video in the DigitizeIt software page [4] gives a detailed description of this step. Apply this step to each jpeg file to obtain 6 text files.

**Step 3: Reconstructing patient-level data**

**Fig. 8:**
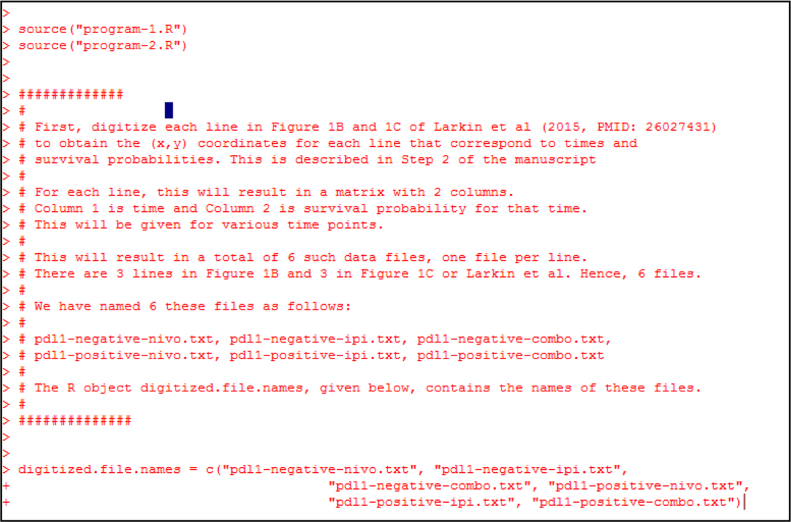
First, read the two programs “program-1.R” and “program-2.R” using the “source” command in R. Here “program-1.R” contains the R function “preprocess.digitized.data” to perform the pre-processing step, and “program-2.R” contains the R function “Guyot.individual.data” that performs survival probability inversion steps described by Guyot et al. [6] to reconstruct patient-level data. These functions can be downloaded from https://www.mskcc.org/sites/default/files/node/137932/documents/2017-04-20-14-31-36/dataexample.zip. Next, create an R object “digitized.file.names”, which is a character vector of the names of the text files containing the (x,y) data for the 6 lines. We have named the files as “pdl1-negative-nivo.txt”, “pdl1-negative-ipi.txt” etc.

**Fig. 9:**
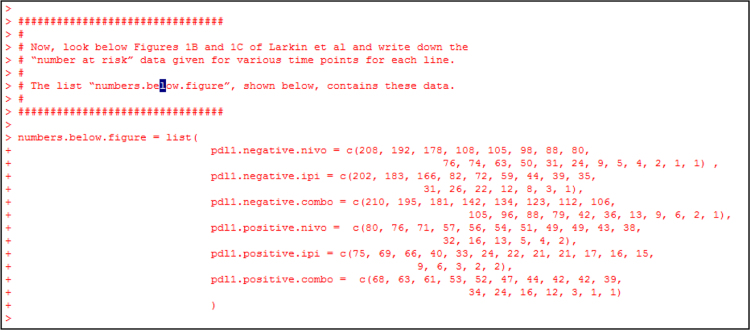
Create an R object “numbers.below.figure” as a list containing 6 elements. Each element is a vector containing the numbers at risk given below Figs. 1B and C of Larkin et al. [2].

**Fig. 10:**
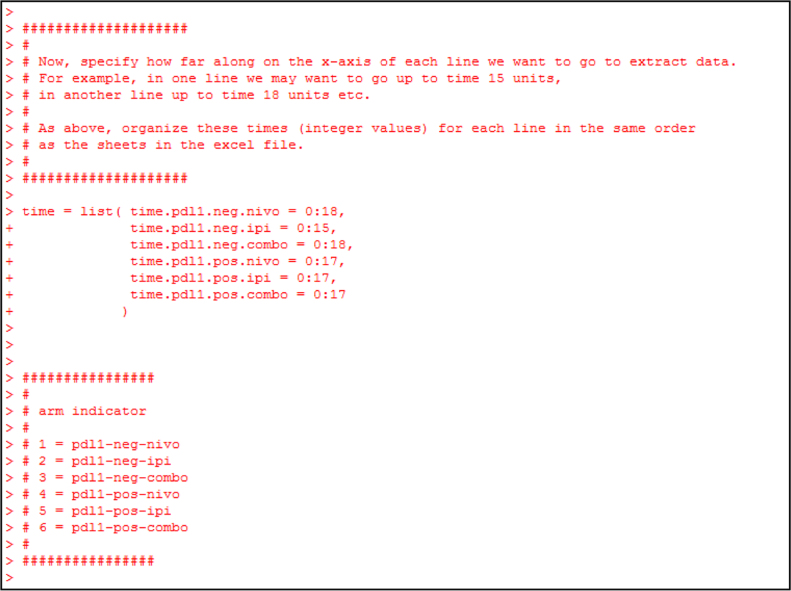
Create an R object “time” as a list containing 6 vectors. Each vector is a set of integers giving the time points along the x-axis of Figs. 1B and C of Larkin et al. [2]. The commented items referred to as “arm indicator” denote the treatment/biomarker arm. This is a simple book-keeping strategy for the user to note that the first file to be digitized corresponds to data from patients with negative programmed death 1 ligand expression receiving nivolumab (denoted “pdl1.neg.nivo”), the second file corresponds to negative programmed death 1 ligand expression receiving ipilimumab (denoted “pdl1-neg-ipi”) etc.

**Fig. 11:**
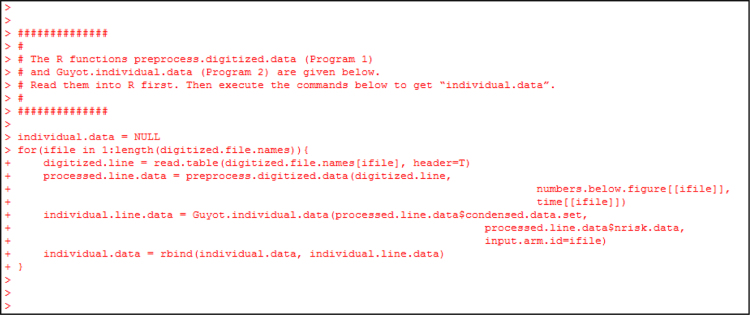
The R object “individual.data” will contain the patient-level digitized data. This object is assembled by running the functions preprocess.digitized.data (in program-1.R) and Guyot.individual.data (in program-2.R) using the (x,y) data sets corresponding to each of the 6 digitized lines. The “for” loop runs these functions for each (x,y) data set.

**Fig. 12:**
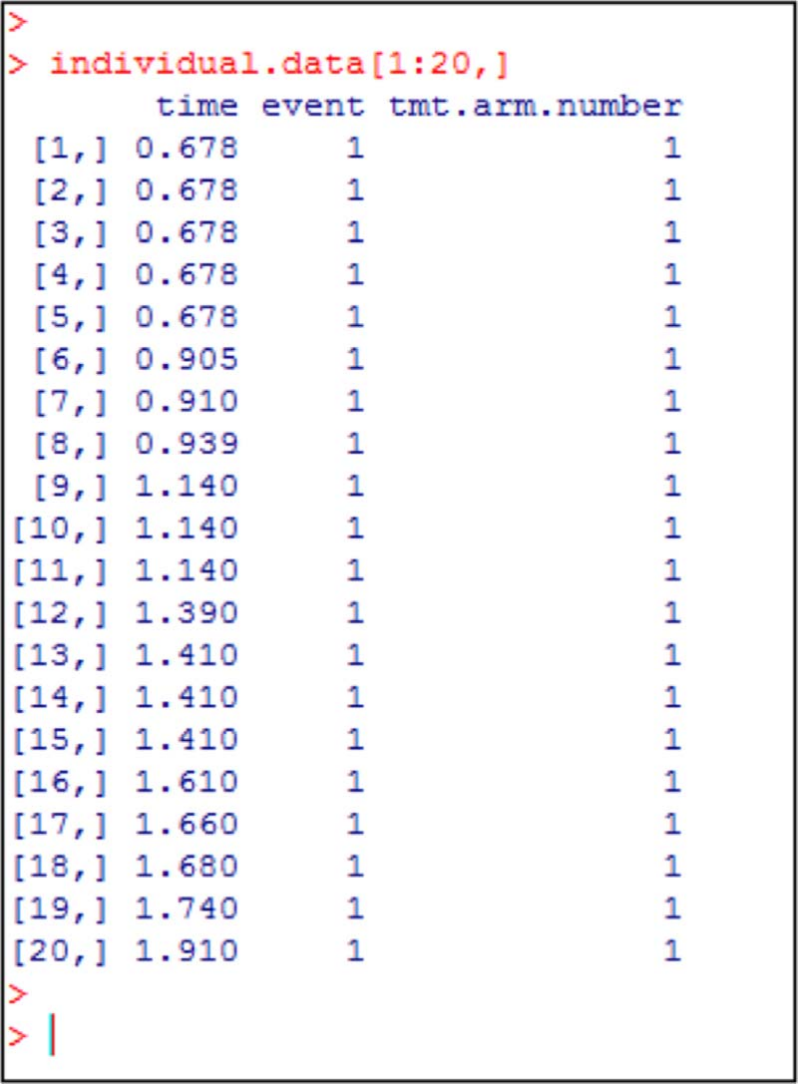
R output showing the first 20 rows of the digitized patient level data. These are the first 20 rows of the object “individual.data”. Column 1 gives the progression free survival time, Column 2 is the event status (1 = disease progression, 0 = no progression). Column 3 is treatment arm number indicating the treatment/biomarker arm, which takes values 1, 2, 3, 4, 5 or 6 (see Fig. 10). These first 20 patients have treatment arm number as 1 in Column 3 since these are patients with negative programmed death 1 ligand expression receiving nivolumab treatment. The data for all the 843 patients can be downloaded from https://www.mskcc.org/sites/default/files/node/137932/documents/2017-04-20-14-31-36/dataexample.zip.

**Fig. 13:**
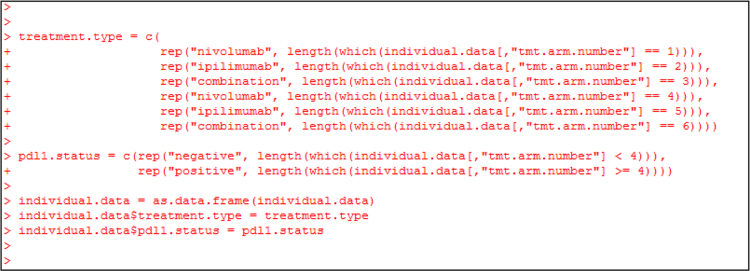
R commands to convert the treatment arm indicator numbers 1, 2, 3, 4, 5, 6 to treatment names (“nivolumab”, “ipilimumab” and “combination”) and programmed death 1 ligand status (“negative” and “positive”), and to append columns for treatment names and expression status to the patient-level data object “individual.data”.

**Fig. 14:**
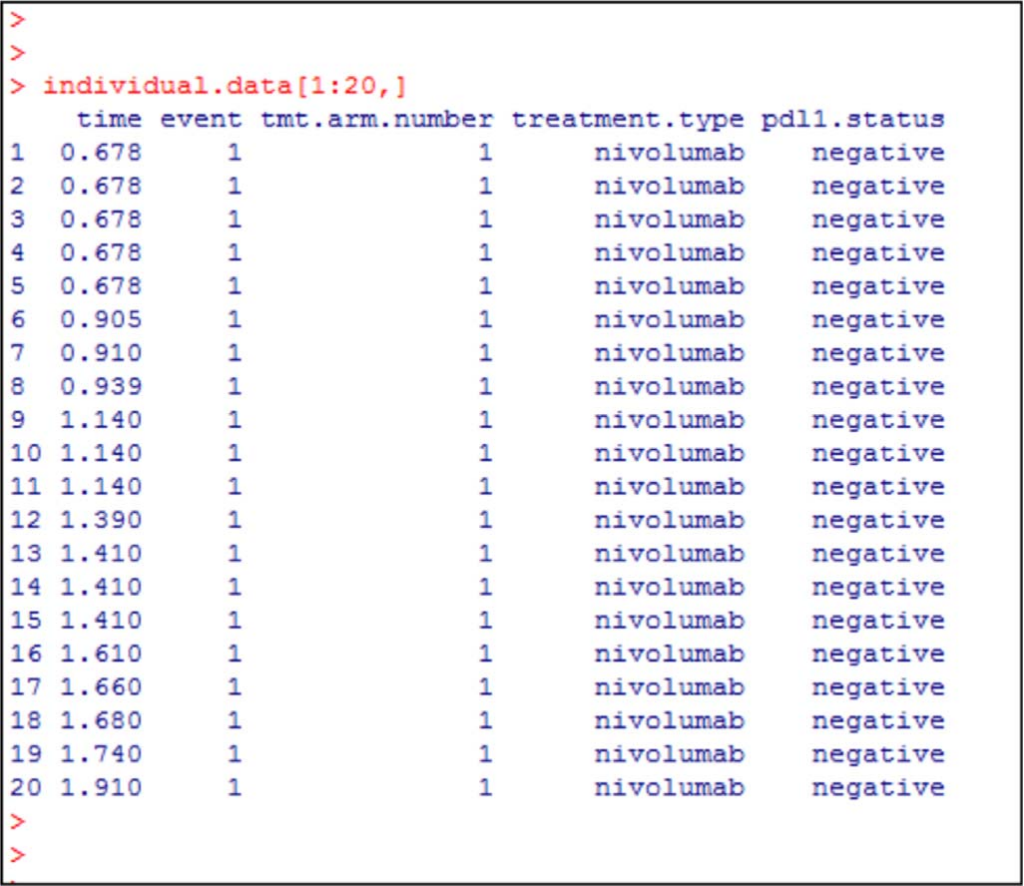
R output showing reconstructed patient-level data for the first 20 patients. The first 3 columns are the same as in Fig. 12. Columns 4 and 5 are the newly appended data on treatment and programmed death 1 ligand expression status using the commands shown in Fig. 13. The data for all 843 patients are given in https://www.mskcc.org/sites/default/files/node/137932/documents/2017-04-20-14-31-36/dataexample.zip.

To obtain patient-level data, first pre-process the (x,y) values corresponding to each line obtained in Step 2 using Program 1. Next, use these parameters as the input for Program 2, which is an R function written by Guyot et al. [6], to obtain the reconstructed patient-level data. These steps are shown in Fig. 8, Fig. 9, Fig. 10, Fig. 11, Fig. 12, Fig. 13, Fig. 14.

## Funding sources

This work was supported by research grants R01 CA137420, R01 CA197402 and P30 CA008748 from the National Cancer Institute, USA, and grant UL1RR024996 from the Clinical and Translational Science Center at Weill Cornell Medical College, New York, USA. The content is solely the responsibility of the authors and does not necessarily represent the official views of the National Institutes of Health.
