# Supplementary material for: A reconstructed melanoma data set for evaluating differential treatment benefit according to biomarker subgroups
Source: Data Brief. 2017 May 5;12:667–75. doi: 10.1016/j.dib.2017.05.005 (PMC5435579; doi:10.1016/j.dib.2017.05.005)
Supplement: Supplementary file 1 — Supplementary material [file mmc1.docx]

Author Conflict of Interest form

**Manuscript Title:** A reconstructed melanoma data set for evaluating differential treatment benefit according to biomarker subgroups

**Authors:** Jaya M. Satagopan, Alexia Iasonos, Joseph G. Kanik

**Journal name:** Data in Brief

**CONFLICT OF INTEREST:** NONE
