# Supplementary material for: A reconstructed melanoma data set for evaluating differential treatment benefit according to biomarker subgroups
Source: Data Brief. 2017 May 5;12:667–75. doi: 10.1016/j.dib.2017.05.005 (PMC5435579; doi:10.1016/j.dib.2017.05.005)
Supplement: Supplementary file 3 — Supplementary material [file mmc3.rtf]

Supplementary Material
All the R computer programs, the digitally extracted (x,y) coordinates for the lines in Figures 1B and 1C of Larkin et al (2015) [2], and the digitally reconstructed patient-level data for all 843 patients can be downloaded from https://www.mskcc.org/sites/default/files/node/137932/documents/2017-04-20-14-31-36/dataexample.zip. 
